# Supplementary material for: Deficiency in FTSJ1 Affects Neuronal Plasticity in the Hippocampal Formation of Mice
Source: Biology (Basel). 2022 Jul 5;11(7):1011. doi: 10.3390/biology11071011 (PMC9312013; doi:10.3390/biology11071011)
Supplement: Supplementary file 1 [file biology-11-01011-s001.zip › biology-1779522-supplementary.pdf]

Table S1:

A: LC-MS/MS parameter (data independent mode; quantitative data).

Data independent analyses (DIA)

reversed phase liquid chromatography	**Ultimate 3000 RSLC (Thermo Scientific)**
Trap column	75 µm inner diameter, packed with 3 µm C18 particles (Acclaim PepMap100, Thermo Scientific)
Analytical column	75 µm inner diameter, packed with 2.6 µm C18 particles (Accucore, 25 cm, Thermo Scientific)
Flow rate	300 nl/min
column oven temperature	40°C
buffer system	binary buffer system consisting of 0.1% acetic acid in HPLC-grade water (buffer A) and 100% ACN in 0.1% acetic acid (buffer B)
gradient	gradient of buffer B: 2min 2% to 5 %, 8min 5%, 120min 5% to 25%, 5min 25 to 40%, 2 min 40% to 90%, 5 min 90%, 3 min 90% to 2%, 5 min 2%
Mass spectrometer	**Q Exactive HF**
operation mode	data-independent
electrospray	Nanospray Flex Ion Source
Full MS	
MS scan resolution	60,000
AGC target	5e6
maximum ion injection time for the MS scan	200 ms
Scan range	333 to 1650 *m/z*
Spectra data type	profile
dd-MS2	
Resolution	30,000
MS/MS AGC target	3e6
maximum ion injection time for the MS/MS scans	auto
Spectra data type	profile
selection for MS/MS	1
isolation window	56 windows *m/z* 13
Fixed first mass	200
dissociation mode	higher energy collisional dissociation (HCD)
normalized collision energy	stepped, 27.5

dissociation mode	HCD

B: Spectronaut parameters for peptide/Protein identification and intensity extraction

**Spectronaut 15.7.220308.50606**	
Computer Name: AGVOE-SPECTRONA	
User Domain Name: AGVOE-SPECTRONA	
User Name: spectronaut	
Analysis Mode: UI	
Analysis Type: directDIA	
Analysis Date: 23-March-2022 10:05:27 UTC+1	
Settings Used:	
**Pulsar Search\Peptides**	
Toggle N-terminal M:	True
Min Peptide Length:	7
Max Peptide Length:	52
Missed Cleavages:	2
Digest Type:	Specific
Enzymes / Cleavage Rules:	Trypsin/P
**Pulsar Search\Labeling**	
Channels:	
Channel 1:	False
Channel 2:	False
Channel 3:	False
**DIA Analysis\Data Extraction**	
MS1 Mass Tolerance Strategy:	Dynamic
Correction Factor:	1
MS2 Mass Tolerance Strategy:	Dynamic
Correction Factor:	1
Intensity Extraction MS1:	Maximum Intensity
Intensity Extraction MS2:	Maximum Intensity
**DIA Analysis\XIC Extraction**	
XIC IM Extraction Window:	Dynamic
Correction Factor:	1
XIC RT Extraction Window:	Dynamic
Correction Factor:	1
**Pulsar Search\Modifications**	
Max Variable Modifications:	5
**Database**	
Original File:	mouse_uniprot_20210221.fasta
**Select Modifications:**	
Fixed Modifications::	Carbamidomethyl (C)
Variable Modifications: :	Acetyl (Protein N-term), Oxidation (M)
**DIA Analysis\Calibration**	

MS1 Mass Tolerance Strategy:	System Default
MS2 Mass Tolerance Strategy:	System Default
Precision iRT:	True
iRT <-> RT Regression Type:	Local (Non-Linear) Regression
Exclude Deamidated Peptides:	True
MZ Extraction Strategy:	Maximum Intensity
Allow source specific iRT Calibration:	True
**DIA Analysis\Identification**	
Generate Decoys:	True
Decoy Limit Strategy:	Dynamic
Library Size Fraction:	0.1
Decoy Method:	Mutated
Preferred Fragment Source:	NN Predicted Fragments
Machine Learning:	Per Run
Exclude Duplicate Assays:	True
Precursor PEP Cutoff:	0.2
Protein Qvalue Cutoff (Experiment):	0.01
Protein Qvalue Cutoff (Run):	0.05
Exclude Single Hit Proteins:	False
Pvalue Estimator:	Kernel Density Estimator
Precursor Qvalue Cutoff:	0.001
Single Hit Definition:	By Stripped Sequence
**DIA Analysis\Quantification**	
Interference Correction:	True
MS1 Min:	2
MS2 Min:	3
Exclude All Multi-Channel Interferences:	True
Only Identified Peptides:	True
Protein LFQ Method:	Automatic
Major (Protein) Grouping:	by Protein Group Id
Minor (Peptide) Grouping:	by Stripped Sequence
Minor Group Top N:	False
Minor Group Quantity:	Sum precursor quantity
Major Group Top N:	True
Min:	2
Max:	3
Major Group Quantity:	Mean peptide quantity
Quantity MS-Level:	MS2
Quantity Type:	Area
Proteotypicity Filter:	None (set to proteotypic by in-house analysis R pipeline)
Data Filtering:	Qvalue sparse
Fraction:	NA
Imputing Strategy:	No Imputing
Cross Run Normalization:	True (local)

**DIA Analysis\PTM Workflow**	
PTM Localization:	True
Probability Cutoff:	0.75
PTM Analysis:	True
Multiplicity:	True
Run Clustering:	False
PTM Consolidation:	Sum
Flanking Region:	7
**DIA Analysis\Workflow**	
MS2 DeMultiplexing:	Automatic
Run Limit for directDIA Library:	-1
Method Evaluation:	False
Profiling Strategy:	iRT Profiling
Profiling Row Selection:	Minimum Qvalue Row Selection
Qvalue Threshold:	0.001
Profiling Target Selection:	Profile only non-identfied Precursor
Identification Criterion:	Qvalue
Threshold:	0.001
Carry-over exact Peak Boundaries:	False
Unify Peptide Peaks Strategy:	Select corresponding Peak
**DIA Analysis\Protein Inference**	
Protein Inference Workflow:	Automatic
Inference Algorithm:	IDPicker
**DIA Analysis\Post Analysis**	
Calculate Sample Correlation Matrix:	True
Calculate Explained TIC:	Quick
Differential Abundance Grouping:	Major Group (Quantification Settings)
Smallest Quantitative Unit:	Precursor Ion (Quantification Settings)
Use All MS-Level Quantities:	False
Differential Abundance Testing:	Paired t-test
Group-Wise Testing Correction:	False
Run Clustering:	True
Distance Metric:	Manhattan Distance
Linkage Strategy:	Ward's Method
Z-score transformation:	False
Order Runs by Clustering:	True
**DIA Analysis\Pipeline Mode**	
Post Analysis Reports:	
Scoring Histograms:	True
Data Completeness Bar Chart:	True
Run Identifications Bar Chart:	True
CV Density Line Chart:	True
CVs Below X Bar Chart:	True
Generate SNE File:	True

Store Iontraces in SNE:	False
Report Schema:	C_FunGene_complex (Normal)
Reporting Unit:	Across Experiment
**Pulsar Search\Identification**	
Peptide FDR:	0.01
Protein Group FDR:	0.01
PSM FDR:	0.01
**Pulsar Search\Tolerances**	
Tolerance Parameters:	
Thermo Orbitrap:	
Calibration Search:	Dynamic
MS1 Correction Factor:	1
MS2 Correction Factor:	1
Main Search:	Dynamic
MS1 Correction Factor:	1
MS2 Correction Factor:	1
TOF:	
Calibration Search:	Dynamic
MS1 Correction Factor:	1
MS2 Correction Factor:	1
Main Search:	Dynamic
MS1 Correction Factor:	1
MS2 Correction Factor:	1
Thermo IonTrap:	
Calibration Search:	Dynamic
MS1 Correction Factor:	1
MS2 Correction Factor:	1
Main Search:	Dynamic
MS1 Correction Factor:	1
MS2 Correction Factor:	1
**Pulsar Search\Workflow**	
Use DNN Predicted Ion Mobility:	Auto
Fragment Ion Selection Strategy:	Intensity Based
In-Silico Generate Missing Channels:	False
**Pulsar Search\Result Filters**	
Precursors:	
Best N Fragments per Peptide:	True
Min:	6
Max:	10
Channel Count:	False
Modifications:	None
Amino Acids:	False
Best N Peptides per Protein Group:	False
FASTA Matched:	False

Missed Cleavage:	False
Peptide Charge:	False
Proteotypicity:	False
Fragment Ions:	
m/z :	True
Min:	300
Max:	1800
Ion Charge:	False
Ion Loss Type:	False
Ion Type:	False
Ion AA Length:	True
N:	3
Relative Intensity:	True
Min:	5

Table S2:

Differentially expressed proteins identified by mass spectrometry.

gene	UniProt/ Swiss-Prot Accession	entry-name	Entrez Gene Name	gene name	Expr Fold Change	Expr p-value
FXYD6	Q9D164	FXYD6	FXYD domain containing ion transport regulator 6	Fxyd6	3.39	1.68E-02
KRT5	Q922U2	K2C5	keratin 5	Krt5	3.164	1.22E-02
DSP	E9Q557	DESP	desmoplakin	Dsp	2.35	1.35E-02
ANXA2	P07356	ANXA2	annexin A2	Anxa2	2.325	1.40E-03
ATP2C1	Q80XR2	AT2C1	ATPase secretory pathway Ca2+ transporting 1	Atp2c1	1.885	3.12E-02
JUP	Q02257	PLAK	junction plakoglobin	Jup	1.865	4.13E-02
NECTIN1	Q9JKF6	NECT1	nectin cell adhesion molecule 1	Nectin1	1.545	2.00E-03
CTSB	P10605	CATB	cathepsin B	Ctsb	1.457	8.50E-03
MBP	P04370	MBP	myelin basic protein	Mbp	1.398	1.07E-02
MAG	P20917	MAG	myelin associated glycoprotein	Mag	1.39	2.87E-02
NDUFB9	Q9CQJ8	NDUB9	NADH:ubiquinone oxidoreductase subunit B9	Ndufb9	1.38	9.30E-03
BRK1	Q91VR8	BRK1	BRICK1 subunit of SCAR/WAVE actin nucleating complex	Brk1	1.363	4.99E-02
NUDT16L1	Q8VHN8	TIRR	nudix hydrolase 16 like 1	Nudt16l1	1.356	3.84E-02
GAP43	P06837	NEUM	growth associated protein 43	Gap43	1.352	4.80E-03

ATP1B3	P97370	AT1B3	ATPase Na+/K+ transporting subunit beta 3	Atp1b3	1.335	1.16E-02
VCAM1	P29533	VCAM1	vascular cell adhesion molecule 1	Vcam1	1.321	4.48E-02
NUDT2	P56380	AP4A	nudix hydrolase 2	Nudt2	1.315	4.75E-02
RAB22A	P35285	RB22A	RAB22A. member RAS oncogene family	Rab22a	1.308	1.41E-02
LXN	P70202	LXN	latexin	Lxn	1.306	2.05E-02
LARS1	Q8BMJ2	SYLC	leucyl-tRNA synthetase 1	Lars1	-1.304	1.31E-02
ZFR	O88532	ZFR	zinc finger RNA binding protein	Zfr	-1.307	3.40E-03
PAFAH1B2	Q61206	PA1B2	platelet activating factor acetylhydrolase 1b catalytic subunit 2	Pafah1b2	-1.311	0.00E+00
KHDRBS3	Q9R226	KHDR3	KH RNA binding domain containing. signal transduction associated 3	Khdrbs3	-1.311	3.40E-03
PPP1R13B	Q62415	ASPP1	protein phosphatase 1 regulatory subunit 13B	Ppp1r13b	-1.312	4.90E-03
VPS33A	Q9D2N9	VP33A	VPS33A core subunit of CORVET and HOPS complexes	Vps33a	-1.466	3.88E-02
TRAPPC12	Q8K2L8	TPC12	trafficking protein particle complex subunit 12	Trappc12	-1.553	4.65E-02
PDPR	Q7TSQ8	PDPR	pyruvate dehydrogenase phosphatase regulatory subunit	Pdpr	-1.585	1.29E-02
